# A mixed-methods needs assessment of adult diabetes mellitus (type II) and hypertension care in Toledo, Belize

**DOI:** 10.1186/s12913-017-2075-9

**Published:** 2017-02-28

**Authors:** Annette M. Dekker, Ashley E. Amick, Cecilia Scholcoff, Ashti Doobay-Persaud

**Affiliations:** 1 Feinberg School of Medicine, Northwestern University, 420 East Superior Street, Chicago, IL 60611 USA; 2 Medical College of Wisconsin, Milwaukee, WI USA

**Keywords:** Type 2 diabetes, Hypertension, Non-communicable disease, Mixed method, Needs assessment, Central America

## Abstract

**Background:**

Non-communicable diseases, including diabetes mellitus and hypertension, continue to disproportionately burden low- and middle-income countries. However, little research has been done to establish current practices and management of chronic disease in these settings. The objective of this study was to examine current clinical management and identify potential gaps in care of patients with diabetes mellitus and hypertension in the district of Toledo, Belize.

**Methods:**

The study used a mixed methodology to assess current practices and identify gaps in diabetes mellitus and hypertension care. One hundred and twenty charts of the general clinic population were reviewed to establish disease epidemiology. One hundred and seventy-eight diabetic and hypertensive charts were reviewed to assess current practices. Twenty providers completed questionnaires regarding diabetes mellitus and hypertension management. Twenty-five individuals with diabetes mellitus and/or hypertension answered a questionnaire and in-depth interview.

**Results:**

The prevalence of diabetes mellitus and hypertension was 12%. Approximately 51% (*n* = 43) of patients with hypertension were at blood pressure goal and 26% (*n* = 21) diabetic patients were at glycemic goal based on current guidelines. Of the patients with uncontrolled diabetes, 49% (*n* = 29) were on two oral agents and only 10% (*n* = 6) were on insulin. Providers stated that barriers to appropriate management include concerns prescribing insulin and patient health literacy. Patients demonstrated a general understanding of the concept of chronic illness, however lacked specific knowledge regarding disease processes and self-management strategies.

**Conclusions:**

This study provides an initial overview of diabetes mellitus and hypertension management in a diverse patient population in rural Belize. Results indicate areas for future investigation and possible intervention, including barriers to insulin use and opportunities for lifestyle-specific disease education for patients.

## Background

The global prevalence of non-communicable disease continues to increase with a disproportionate burden placed on low- and middle-income countries. Of the 350 million people suffering from diabetes mellitus (type II), nearly 80% live in low-and middle-income countries [1]. While chronic disease accounts for 40% of worldwide deaths from all causes, this percentage rises to 80% of deaths in low- and middle-income countries [2]. The pattern of prevalence and mortality is consistent in Belize, a middle-income country whose prevalence of diabetes mellitus (DM) and hypertension (HTN) has reached 13.1 and 28.7%, respectively [3]. In the last decade, diabetes mellitus and hypertension have emerged as the first and second leading causes of mortality in the country [3]. In addition to increasing mortality, chronic disease causes a catastrophic loss in economic production amounting to trillions of dollars per year [4].

Despite the high mortality and associated economic burden, there is a paucity of evidence establishing chronic disease prevalence and management in the setting of low- and middle-income countries [4, 5]. The emphasis of research on non-communicable diseases has been focused on high-income countries. In recognition of this unmet need, this descriptive study aims to highlight chronic disease care in Toledo, Belize with the use of a multidimensional methodology to more fully capture the current environment and identify gaps in care.

## Methods

### Objective

The aim of this study was to broadly define the current process by which care is delivered and identify barriers and gaps in the current management of adults with diabetes mellitus and hypertension in Toledo, Belize. The objectives of the study were to define 1) the prevalence of DM and HTN in the clinic population; 2) the current state of care and disease control; 3) provider practice patterns and perspectives on treating DM and HTN in this setting; and 4) patients understanding of chronic illness and their experiences with managing their disease.

### Study setting

The study was conducted at Hillside Health Care International (HHCI) in Toledo, Belize. The Toledo district is comprised of many diverse ethnic groups including Mayan, Garifuna, Creole, East Indian, Mestizo, Chinese, and Mennonite. Indigenous Mayan populations represent a majority in this region, and speak primarily Q’eqchi’ or Mopan Maya [6]. Toledo is the poorest district in Belize and 79% of the population lives below the poverty line with an estimated household income of 1400 USD per year [7]. Although the literacy rate is 75%, only 11% complete secondary school [7].

**Fig. 1:**
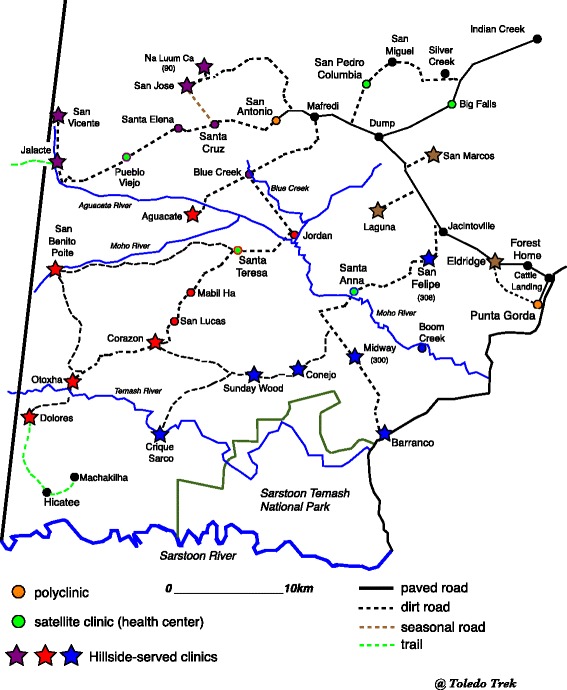
Hillside Clinic and Mobile Sites in Toledo District

HHCI is a non-profit organization established in 2000 to provide primary care for adults and children. Services are provided at the freestanding clinic in Eldridge, Toledo, as well as 16 mobile clinic sites that are visited on a regular schedule (Fig. 1). The clinic includes a pharmacy that provides available medications free of charge. In total, HHCI provides approximately 10,000 clinic visit per year. HHCI is staffed by a multidisciplinary provider cohort. Providers are both local and international, and have either short or long term commitments. HHCI is actively engaged in medical education and public health, and hosts rotating medical, pharmacy, physician assistant, nursing, physical therapy, and public health students.

### Study design

**Fig. 2:**
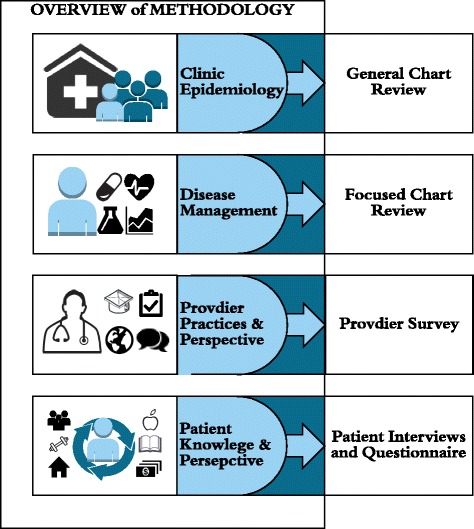
Overview of Methodology

This study utilized a mixed-methods approach. Components of the study included a general chart review of adult patients, a focused chart review of all known patients with diabetes mellitus and hypertension, a provider survey, and in-depth patient interviews and questionnaire (Fig. 2). Research was conducted between June 1^st^, 2013 and August 1^st^, 2015. The research methodology was reviewed and approved by the Northwestern University Institutional Review Board as well as the Belize Ministry of Health.

#### Epidemiology of clinic population: general chart review

Two general chart reviews of 60 charts each were conducted to determine baseline characteristics of the patient population and to estimate the prevalence of DM and HTN at HHCI. The initial chart review was conducted in June 2014. Charts stored at Hillside Clinic were chosen through random selection of 1–20 cabinets, followed by random selection of one third of the cabinet, and selection of a chart from the designated third to establish population demographics, disease prevalence, and screening prevalence. The chart review was repeated in January 2015 using the same technique to confirm the previous disease prevalence estimates at a second time point and obtain additional information regarding obesity and screening practices (not included here). Inclusion criteria for both reviews were non-pregnant adults ≥18 years of age. Patients were considered to have diabetes mellitus or hypertension as labeled in the chart under patient diagnoses.

#### Current practice and quality of care: focused chart review

A comprehensive chart review was conducted of every patient who had been diagnosed with HTN and/or DM at HHCI the time of the study. A total of 178 charts were reviewed. Data extracted included demographic information, laboratory data, therapeutic interventions, documented lifestyle counseling, and evidence of end organ dysfunction. Inclusion criteria were non-pregnant adults ≥18 years of age with a diagnosis of hypertension or diabetes in the chart prior to June 2014. JNC8 guidelines were used for hypertensive guidelines as they were most current at the time of the study, and control was more liberally defined than prior guidelines. In 2014–2015 the Belize MOH guidelines were more rigorous and consistent with the JNC7 guidelines [8–10].

#### Provider practice pattern and perspectives: online survey

A provider survey was conducted to better define provider demographics, practice patterns, and perceived barriers to managing DM and HTN at HHCI. The survey was adapted from prior published provider surveys of LMIC proviers [11], and constructed in SurveyGizmo. Providers were eligible if they had served in a supervisory role at HHCI from 2013 to 2014. The survey was open to physicians, nurse practitioners, physician assistants, and pharmacists. Authors were provided a contact list by HHCI leadership, and all individuals were sent the online survey via the email address provided.

#### Patient perspectives: Structured interviews and questionnaire

In depth interviews were conducted with non-pregnant adult patients (≥18 years of age) diagnosed with diabetes mellitus and/or hypertension. Interviews were conducted in English, Q’eqchi’ Mayan, or Mopan Mayan by a female researcher and translator. Individuals were assured that responses would be anonymized and that they could decline to participate, decline to answer any specific questions, or end the interview at any time without negative consequence. All individuals who participated were asked to provide oral consent.

Patient interviews consisted of a structured interview as well as a questionnaire. The structured interview focused on general themes of health and sickness, as well as specific knowledge of diabetes and hypertension [12, 13]. Open-ended questions included items such as “what causes sickness?” and “what is diabetes?” The questionnaire included 51 close-ended questions focused on socio-demographics, risk factors for diabetes and hypertension as well as management of diabetes and hypertension. Quantitative questions were adapted from the 2009 Central America Diabetes Initiative (CAMDI) Survey of Diabetes, Hypertension and Chronic Disease Risk Factors: Belize [3].

### Data analysis

Quantitative data, including chart review, patient questionnaires, and provider survey were entered using Microsoft Excel 2011 (version 14.3.6; Microsoft Corporation, Redmond, WA) and imported to Stata Data Analysis and Statistical Software (version 10,1; Stata Corporation, College Station, TX). Key variables were analyzed for frequency and/or mean and standard deviation. Missing or unavailable data was a frequent occurrence due to limited access to laboratory services and variable charting practices. Patients with missing data were eliminated from analysis for the particular variable in question. Analysis of NCD control (glucose levels for DM, blood pressure for HTN) was limited to patients who had visited the clinic within the past 12 months, and had data for the variable in question recorded in the chart. Qualitative interviews with patients were audio recorded with the consent of participants. Data were transcribed and hand-coded based on key themes identified through a content analysis. Subsequent analyses identified emergent themes and explored consistency among responses [14].

## Results

### Epidemiology of clinic population: General chart reviews

The initial chart review in June 2014 demonstrated that the majority of patients were female (65%) and Mayan (64%) or East Indian (17%) (Table 1). The average age was 44 (±17) years. Patients visited the clinic 2.1 times per year on average. The prevalence of diabetes was 12% and the prevalence of hypertension was also 12%. In January 2015, a second sample of 60 charts demonstrated similar findings with the prevalence of both DM and HTN at 13%.

**Table 1:** Demographics of overall patient population provided care at clinic

Chart review	I	II
Demographic	*n* = 60	*n* = 60
Female – % (*n* = 60)	62	75
Age – yrs (*n* = 60)	44 ± 17	42 ± 15
Ethnicity – %	(*n* = 52)	(*n* = 60)
Maya	64	70
Garifuna	4	8
East Asian/Indo-carribean	17	2
Creole/Afro-caribbean	2	0
Mestizo	0	3
White	0	0
Other	13	18
Clinic visits per year – visits (*n* = 60)	2.1 ± 2	1.9 ± 2.7
Chronic disease – % (*n* = 60)^a^		
Diabetes	12	13
Hypertension	12	13

^a^Documented in problem list or progress notes

### Current practice and quality of care: Focused chart review

One hundred seventy-eight diabetic and hypertensive labeled charts were reviewed, representing all known diabetic and hypertensive patients in the clinic population. One hundred and twelve patients had diabetes and 110 had hypertension (Table 2). Diabetic patients were predominately female and Mayan, with an average age of 52 years. Hypertensive patients were predominately female and East Indian, with an average age of 57 years.

**Table 2:** Demographics of patients with diabetes and/or hypertension identified by chart review

Chart review III		
Demographic data	Diabetes	Hypertension
	*n* = 112	*n* = 110
Female	74%	73%
Age – yrs	52 ± 14	59 ± 14
Ethnicity		
East Asian/Indo-carribean	22%	32%
Garifuna	10%	16%
Mayan	41%	12%
Creole/Afro-caribbean	7%	11%
Mestizo	3%	4%
White	3%	4%
Other	24%	21%
Clinic visits per year – visits	3.4 ± 4	4.1 ± 4.2

Diabetic patients (*n* = 112) were on 1.3 oral medications on average and 9% (*n* = 10) were on insulin therapy (Table 3). Medications used included metformin, sulfonylureas, and NPH insulin. Further analysis was limited to patients with at least one clinic visit within 12 months prior to study onset to obtain information about the current practices and standards of care at HHCI. Eighty-three of the 112 diabetics had visited the clinic in the past 12 months (*n* = 83, 74%), and of those patients 96% (*n* = 80) had any recorded glucose reading (FBG, RBG). Of the 80 diabetics who had visited in the last year, 50% (*n* = 40) had an available fasting blood glucose (FBG) and 50% (*n* = 40) had a random blood glucose (RBG). The FBG average was 201 (±81, *n* = 40) and the random blood glucose (RBG) average was 255 (±139, *n* = 40). Eighteen percent (*n* = 14) of patients came to clinic with a glucose level (fasting or random) above 300 and 10% had a reading over 400. Of diabetics seen in past 12 months (*n* = 80), only 26% (*n* = 21) of patients were at goal for diabetic control as defined according to current guidelines [15–19].

**Table 3:** Treatment and management of diabetes as identified by chart review III

Chart review III	
Diabetic patients (at least one clinic visit within the past 12 months)	
Diabetic management	*n* = 83
Number of oral diabetic medications^a^ – Num	1.3 ± .7
Patients on insulin therapy	10%
Foot exam in past 12months	41%
Eye referral in past 12months	41%
Serum creatinine in past 12months	39%
Urinalysis in past 12months	28%
Lipid panel ever preformed	67%
Dietary counseling	60%
Diabetic control^b^	*n* = 80
Average fasting blood sugar^b^ (*N* = 40)	201 ± 81
Average random blood sugar^b^ (*N* = 40)	255 ± 139
Diabetics with last BS > 300 - % (*n* = 80)	18%
Diabetics with last BS > 400 - % (*n* = 80)	10%
Diabetics with last BS > 500 - % (*n* = 80)	4%
Diabetics at goal^c^ - % (*n* = 80)	26%

^a^Diabetic medications prescribed include metformin and sulfonylureas

^b^Calculated in patients with FBS or RBS recorded

^c^Based upon guidelines from ADA 2014

Among diabetics seen within the last 12 months (*n* = 80), 74% (*n* = 59) were considered to be uncontrolled according to current guidelines. Among uncontrolled diabetics, 7% (*n* = 4) were on no therapy, 34% (*n* = 20) were on one oral medication, 49% (*n* = 29) were on two oral medications, and 10% (*n* = 6) were on insulin (Table 4). The average FBG in uncontrolled diabetics was 219 (±74) and the average RBG was 333 (±118). Of the few patients on insulin, the majority was Mayan and resided in Punta Gorda, the major urban center of Toledo.

**Table 4:** Management of uncontrolled^a^ diabetic patients (clinic visit in the past 12 months)

Chart review III	
Diabetic management	*N* = 59
Patients on no diabetic therapy	7%
Patients on only one oral medication^b^	34%
Patient on two oral agents^b^	49%
Patients on NPH insulin therapy	10%
Diabetic control	
Average fasting blood glucose (FBG)^c^	219 ± 74
Average random blood glucose (RBG)^c^	333 ± 118

^a^Based upon guidelines from ADA 2014

^b^Diabetic oral medications prescribed include Metformin and Sulfonylureas

^c^Calculated in patients with FBG or RBG recorded

A chart review of all diabetic patients seen in the last year (*n* = 83) revealed that 60% of patient received lifestyle modification counseling, and a minority received routine monitoring for diabetic complications, including foot exams (41%), eye referrals (41%), serum creatinine (39%) and urinalysis (28%).

Of all hypertensive patients identified (*n* = 110), 77% (*n* = 85) had been to HHCI in the past 12 months. Among patients seen in the last year, the average blood pressure on last visit was 141 systolic (±19) and 78 diastolic (±13). Hypertensive patients were on an average of 1.3 (±0.9) antihypertensive medications of various classes, which included ACE inhibitors, calcium channel blockers, beta blockers, and diuretics (Table 5). Fifty-one percent (*n* = 41) of hypertensive patients seen in the last 12 months were at goal blood pressure control as defined by the JNC8 guideline, as previously discussed. Twenty-seven percent of hypertensive patients had documented end-organ damage related to their hypertension.

**Table 5:** Treatment and management of hypertension as documented by chart review III

Chart review III	
Hypertension (clinic visit in past 12 months)	Total *n* = 85
Antihypertensive medications^a^ – num (*n* = 97)	1.3 ± .9
Patients at blood pressure goal^b^ – % (*n* = 80)	51
Lifestyle counseling documented^c^ – %	60
Patients with related end organ disease^d^ – %	27

^a^Medications include ACE-I, ARB, CCB, BB, diuretic

^b^Based upon JNC7 and KNC8 guidelines

^c^Includes weight loss, diet, exercise, smoking, alcohol cessation

^d^Diseases included CAD, PAD, CKD, CVA, CHF

### Stakeholder perspective: Provider perceptions of care

Twenty providers completed the online survey with a response rate of 38%. Eighty percent of respondents were physicians (MD or DO, *n* = 16)), two were nurse practitioners, one was a physician assistant, and one was a pharmacist (Table 6). The majority of providers (80%, *n* = 16) currently practice in an urban or suburban setting. Eighty percent (*n* = 16) trained in the United States.

**Table 6:** Provider demographics

Provider demographics (*n* = 20)	
Type of provider - % (*n* = 20)	
Physician (MD/DO, internist, pediatrician, family practitioner)	80%
RN/RNP	10%
Physician Assistant	5%
Pharmacist	5%
Typical practice setting -	
Outpatient/Clinic	40%
Inpatient	30%
Emergency/Urgent Care	5%
Other	30%
Primary patient population	
Urban/suburban	80%
Rural	15%
Migrant	5%
Country provider trained	
Australia	5%
UK	10%
Guatemala	5%
USA	80%

The majority of providers (70%, *n* = 14) indicated they had a high degree of comfort caring for patient with diabetes mellitus or hypertension. Seventy percent (*n* = 14) of providers reported routinely using guidelines to manage DM and HTN at HHCI. When providers did use guidelines, there was variability with regards to the source, with 35% (*n* = 7) stating they used international guidelines, 25% (*n* = 5) used guidelines from their country of origin, 15% (*n* = 3) used Belize MOH guidelines, 5% (*n* = 1) used clinic guidelines, and 20% (*n* = 4) used other guidelines.

Ninety percent (*n* = 18) of providers state they routinely educate patients about their disease process, and 95% (*n* = 19) state they counsel patients about the importance of lifestyle modification. On average they allot 7.6 minuets per encounter to patient education and counseling. Providers primarily used general dietary and weight loss recommendations, while fewer tailored their recommendations to include locally available foods or culturally acceptable means of exercise.

The majority of providers (70%, *n* = 14) stated there was insulin available at Hillside, however providers expressed hesitation initiating insulin therapy. Major barriers to insulin use reported by providers included lack of refrigeration, lack of glucose test strips, and fear of hypoglycemia (Table 7). One provider stated “when it [insulin] has been there in the past the barriers include lack of refrigeration, which it requires.” Another stated “in the villages there is no fridge, and no test strips, making monitoring difficult.” One provider described how insulin is used, “There is a lack of blood glucose monitoring – even if a patient is newly starting insulin or very unwell they may get 5 test strips for the first week. For longer term patient they get 1 test strip per month!” Providers reported that the most significant barriers to overall care and patient wellness were poverty, cultural health beliefs, and low health literacy. All providers rated patient’s understanding of disease process as moderate to poor. “I regularly encountered poor understanding from patients, and conflicting health beliefs which lead to poor compliance.”

**Table 7:** Selected quotations from providers

Provider perceived barriers to appropriate care of diabetes, including insulin use
• “When it [insulin] has been there in the past the barriers include lack of refrigeration.”
• “In the villages there is no fridge, and no test strips, making monitoring difficult.”
• “There is a lack of blood glucose monitoring – even if a patient is newly starting insulin or very unwell they may get 5 test strips for the first week. For longer term patient they get 1 strip per month!”
• “I regularly encountered poor understanding from patients, and conflicting health beliefs which lead to poor compliance.”

### Stakeholder perspectives: Patients’ understanding of disease

#### General health and risk factors

In total, 25 interviews were conducted, including 12 interviews at Hillside Clinic, 7 in Punta Gorda, and 6 in rural villages. The response rate was 93%. Seventy-six percent (*n* = 19) of individuals had diabetes and 56% (*n* = 14) had hypertension (Table 8). Among diabetics, 68% (*n* = 13) reported a family history of diabetes, while 64% (*n* = 9) of hypertensive patients reported a family history of high blood pressure. Most individuals do not smoke (94%, *n* = 24) or consume alcohol regularly (86%, *n* = 21). On average, individuals eat fruit and vegetables three times a week and primarily use vegetable oil to cook (68%, *n* = 17). Half of individuals report that they engage in an activity that increases their breathing, including walking or domestic work such as washing, at least once a week (48%, *n* = 12).

**Table 8:** Demographics of individuals interviewed with diabetes and/or hypertension

Demographic	Total *n* = 25
Female – % (*n* = 25)	72
Age – yrs (*n* = 25)	59 ± 19
Ethnicity – % (*n* = 25)	
Maya	28
Garifuna	20
East Asian/Indo-carribean	12
Creole/Afro-caribbean	8
Mestizo	8
White	0
Other	24
Home amenities – % (*n* = 25)	
Electricity	80
Running water	72
Primary school education – % (*n* = 25)	84
Employment – % (*n* = 25)	
Unemployed / retired	40
Homemaker	36
Full time	16
Part time	8
Tobacco use – % (*n* = 25)	4
Alcohol use – % (*n* = 25	16
Chronic disease – % (*n* = 25)	
Diabetes	76
Hypertension	56

#### Understanding of health, diabetes, and hypertension

Individuals perceive health as an absence of disease. In particular, they define health in relation to appropriate nutrition and proper sanitation, as well as functionality such as movement and sight. One woman stated, “To be healthy is to be perfect – no problems. But we can make ourselves healthy. It depends on what we put in our mouth.” Accordingly, many individuals believe that sickness is caused by poor diet, as well as environmental factors such as unclean water or pollution (Table 9). As another woman explained, sickness is caused by “what we eat and what we drink. Too much salt. Too much sweet.” Others explain that sickness presents more acutely following a stressful life event. A Garifuna woman with high blood pressure stated, “I have a son that drowned. I said to myself I was not worrying but it was still in my mind. So from then, I have pressure.” Many individuals, however, remain uncertain what causes disease (20%, *n* = 5).

**Table 9:** Selected quotations from individuals with diabetes and hypertension

Individuals’ understanding of health, diabetes, and hypertension
• “To be healthy is to be perfect – no problems. But we can make ourselves healthy. It depends on what we put in our mouth.” –38 y/o F, mixed ethnicity
• “The environment causes sickness.” -40 y/o M, East Indian
• “It [sickness] is from the air. There are too many pollutions – not like before. But because of that same thing the drinking water is not proper. So they improve it a lot by putting that pump by the side of the road.” -43 y/o F, mixed ethnicity
• Sickness is caused by “what we eat and what we drink. Too much salt. Too much sweet.” -38 y/o F, mixed ethnicity
• “When I cry and cry and cry, then I eat and eat and eat – that’s why I catch the sickness.” -44 y/o F, Q’eqchi' Mayan
• “I have a son that drowned. I said to myself I was not worrying but it was still in my mind. So from there, I have pressure.” – 64 y/o F, Garifuna

Diabetes is most commonly described as “sweet blood.” In general, it is thought that diabetes can be caused by poor nutrition, stress, or genetics. Although some individuals diagnosed with diabetes are able to identify that increased blood glucose is a result of disease affecting the pancreas and insulin (16%, *n* = 3), a minority are able to correctly state signs and symptoms of high blood glucose or complications from diabetes (35%). Eighty-four percent (*n* = 16) have never heard of a hemoglobin A1C.

Individuals with diabetes report that they manage their disease through medication, diet, exercise, and, on occasion, traditional remedies (Table 10). While many individuals state that they try to consume less sugar and eat more vegetables, some are confused what a recommended diet specifically entails. In response to how she has adjusted her diet for her diabetes, one Q’eqchi'  woman explained, “I do not eat much salt or lard. I do not drink any coffee.” Others speak of difficulties maintaining an appropriate diet. “It is hard to eat vegetables everyday. You get tired of it. You need other foods too.”

**Table 10:** Selected quotations from individuals with diabetes and hypertension

How individuals with diabetes and/or hypertension manage their disease
• “We have to control ourselves for what we eat or drink. With the medication, it helps.” – 53 y/o F, mixed ethnicity
• “Exercise most importantly. Take my medication. Do not eat starchy foot! Mostly vegetables. But it is hard to eat veg everyday.” – 38 y/o F, mixed ethnicity
• “I do not eat much salt or lard. I do not drink any coffee.” – 31 y/o F, Q’eqchi' Mayan
• “You have to eat less salt. The thing that you eat – especially when you buy at the shop. It has salt. Like pig tail. It has a lot of salt. I eat it only once a week.” – 59 y/o M, Garifuna
• “I do not drink sugar. I stop drinking coffee. I eat meat. Not fats. I try herbs. I try the bitter one – I do not know which one. It works. I drink at morning, midday, and in the evening. I also drink the noni fruit. Raw onion – it is good for high cholesterol. I also eat garlic. I eat raw garlic.” – 41 y/o F, Q’eqchi' Mayan

Hypertension is less well understood among individuals interviewed. Many individuals with hypertension are unable to explain what high blood pressure is or what causes it. Some perceptions include that hypertension is caused by excessive stress or eating too much salt. In explaining what causes high blood pressure, one Garifuna man said, “It’s salt in your blood. Your blood travels inside your body and it goes to your head. It gives you headaches.”

Similar to diabetes, individuals manage their hypertension through medication, diet, and traditional remedies. Many individuals try to reduce the amount of salt in their meals. A few individuals identified eating less oranges, limiting their coffee intake, or eating garlic as dietary modifications. Traditional remedies used for both diabetes and hypertension include herbs and plants such as soursop leaves and noni fruit.

#### How knowledge about disease and self-management is attained

Information regarding the etiology and treatment of diabetes and hypertension is acquired through a variety of mediums. When asked how they learned about their disease, many individuals explain that they “hear it around” their communities. Individuals also describe relatives, friends, and coworkers who also have diabetes or hypertension as important sources of information (20%, *n* = 5). “I learn it from my ancestors – my aunts and my grandmother. Because everyone has it, I learn it form them.” Others explain that they have only learned about diabetes and/or hypertension from a doctor, nurse, or community health care worker at health care facility after they were diagnosed (28%, *n* = 7). Yet others describe self-initiated education via medical textbooks, radio programs, or the television (16%, *n* = 4). A few individuals with diabetes learned more about their condition through workshops organized by the Ministry of Health (8%, *n* = 2).

#### Barriers to care

Individuals frequently reference limited access due to financial constraints as a barrier to taking care of their chronic disease (60%, *n* = 15). Such limited access includes a lack of transportation for emergency health care, as well as inadequate means for daily access to fruits and vegetables. Only thirty-two percent (*n* = 8) of individuals report that they have enough money to regularly buy fruits and vegetables. One man with both diabetes and high blood pressure who currently lives in Punta Gorda explained,

*“You see what happen in this part of the country – we eat what we can afford. Sometimes you can afford to buy things for the sugar but not all the time. You have to eat what you found. Vegetables are very expensive. You have a time when you cannot buy any vegetables. So you have to find rice or something. People eat what they find. It is not because they want to eat it, but they have to eat. If you don’t eat, you might get sick. You can die! Things are expensive!”*

## Discussion

This study was a mixed methodology to better define the current practices regarding care in a rural primary care clinic in Toledo, Belize. Our study has revealed four major themes: 1) HHCI patients are generally poorly controlled with regards to their chronic conditions, 2) there is likely underutilization of available pharmacological agents, in-part due to provider misconceptions, 3) patients have a general understanding of chronic illness and the role of lifestyle in disease self-management, and 4) specific lifestyle modification counseling and patient education is underutilized.

Diabetic and hypertensive patients are inadequately controlled, placing this population at elevated risk for preventable morbidity and mortality. Inadequate glycemic control was present in 74% of all diabetics, which is higher than estimates in nations such as the US (51%) [20], Germany (40%) [21], Denmark (51%) [22] and Kenya (61%) [23]. However, this finding is on par with similar data from the region where rates of uncontrolled diabetes were found to be 78% in a study of 9 Latin American countries [24] and 76% in Venezuela [25]. In our study, a substantial proportion of uncontrolled diabetic patients were prescribed either one or no oral medication, suggesting that available oral medications may be underutilized. In addition, over half of the uncontrolled diabetics were not at goal on two oral agents.

According to local and international guidelines [9, 10, 15–17], many of these patients may be candidates for escalation to insulin therapy, however only 10% of these patients were on insulin. Underutilization of insulin in developing nations has been described previously in the literature. In Cambodia a study of diabetics found that while most patient were uncontrolled on oral medications, <4% were on insulin [26]. In Latin American, less than 14.5% of diabetics were on insulin despite very poor glycemic control [24]. At HHCI, a majority of providers reported knowing insulin was regularly available at HHCI, however most express strong hesitations regarding its use in this population. Providers cited several reasons for fear of initiation of insulin, such as lack of cold-chain storage, lack of home glucose monitoring, and poor patient understanding.

In our study, physicians frequently cited lack of home refrigeration as a perceived barrier to insulin use. The insulin available at HHCI during the time of study was NPH human insulin isophane suspension. According to the manufacture, opened vials may be stored at room temperature below 86 °F (30 °C) for up to 31 days [27]. Ambient temperatures in the Toledo district range from 50 F to 95 F with an annual average temperature of 25.7 °C (79 °F) [28, 29]. Pharmacologic studies show that there is no reduction in potency when stored at room temperature, where as another shows no more than 14–18% reduction in potency when stored for 28 days at 98.6 °F. [30, 31] These findings suggest that even in a tropical environment, NPH insulin therapy would be feasible without access to refrigeration if dispensed on a monthly basis.

Home glucose monitoring is commonly recommended for diabetic patients, however there is little evidence it improves quality, safety, or is cost-effective [32–35]. In our study, providers reported the lack of home glucose monitoring as a barrier to insulin initiation. Despite this perception, a recent systematic review found that home glucose monitoring did not reduce hypoglycemic events, nor did it guide therapy [32]. Observational studies have shown that patient adherence to home glucose monitoring is low, even in well-resourced settings. An estimated 21–29% of type II diabetics on insulin have never checked their glucose, and only 17–39% check their glucoses on a daily basis [36, 37]. These finding suggest that most providers, even in developed nations, are managing diabetics on insulin without any additional information gained from self-monitored glucose levels.

Providers reported patient understanding was a barrier to disease self-management and wellness. Our study showed that individuals with diabetes and/or hypertension understand the concept of chronic disease. Patients come to the clinic several times a year for maintenance care even if they do not have acute symptoms. Furthermore, they understand the impact of lifestyle on overall health. Individuals explain that a poor diet, such as consuming too much sugar or salt, can lead to chronic diseases such as diabetes and high blood pressure. Despite this baseline understanding, specific knowledge of pathophysiology and explicit dietary management strategies is limited. Many individuals misidentify the impact of particular foods on blood glucose levels or blood pressure readings.

These findings are broadly consistent with similar studies in Latin America [12, 38–40]. In particular, one study that focused on an indigenous population in rural Guatemala similarly found that patients have a general framework for chronic disease understanding, however they lack knowledge of specific biomedical and treatment strategies [40]. This baseline understanding that lifestyle affects health offers the potential for a tailored intervention on disease pathophysiology and culture-appropriate diet recommendations in the Toledo district.

A majority of providers report providing lifestyle modification counseling, allocating a large portion of the encounter to patient education. While 95% of providers reported providing patient education and counseling, it was objectively documented in only 60% of charts, which implies there may have been over-reporting, under-documentation, or both. Despite the frequency with which providers report counseling patients, a minority of patient report receiving their health information from a health professional. Additional research is needed to further understand the impact of physician delivered lifestyle counseling and patient education in this population.

The aim of this research was to identify gaps in care for diabetes and hypertension at a rural community health clinic in Toledo, Belize. Limitations of this study include generalizability due to the single site and small sample sizes, however the findings contribute to the understanding of the local burden of disease. The inability to truly randomize the general chart review may bias results to patient charts that are more easily selected, thus favoring patients with larger files. The results of the focused chart review were limited by frequent missing data points, as availability of basic laboratory testing is often limited in this low resource setting. Furthermore, only half of patients see in the past year had fasting blood glucose levels available. Given that random glucose levels are considered less accurate than fasting blood glucose values, this may limit the accuracy of estimates of glycemic control. The convenience sampling used for interviews with individuals with diabetes and/or hypertension is likely to differ from the overall diabetic and hypertensive population in Toledo, Belize. In particular, it is possible that individuals identified in Punta Gorda and rural villages may be those that health workers assumed would be more willing and able to speak with researchers. The sample only includes those who have received care and thereby potentially neglects individuals who do not seek treatment at health care clinics. Additionally, there was a low survey response-rate, and this may increase the likelihood of non-responder bias. However, despite the low rate it was similar to previously published response rates for online physician surveys [41, 42].

Despite these limitations, the mixed-methodology of this study provides a multidimensional assessment in a region where the current understanding of diabetes mellitus and hypertension management is limited. The open-ended structure of individual interviews further ensured that participants’ responses were not influenced by western biomedical views.

## Conclusion

Mounting evidence supports the rise of morbidity and mortality from non-communicable diseases such as diabetes and hypertension in the developing world. However, little evidence exists regarding the management of patients with these diseases in resource-poor global settings. Obtaining a comprehensive understanding of the current delivery of care and stakeholder perspectives is a fundamental step in improving the quality of care and focusing future interventions. We present a mixed-methods descriptive study aimed at better characterizing the current management of diabetes and hypertension in a primary care clinic in Toledo, Belize. Our findings suggest that overall control of both diabetes and hypertension is poor. While poor control is undoubtedly multifactorial, findings of this preliminary study suggest that underutilization of available pharmacologic interventions and a lack of culture-specific patient counseling are important contributors to ineffective management. This study provides a basis for future investigations to develop strategies for the ever-rising burden of DM and HTN in resource-limited settings.

## Abbreviations

DM: Diabetes mellitus type II
HHCI: Hillside Health Care International
HTN: Hypertension.
